# Teacher's Physical Activity and Mental Health During Lockdown Due to the COVID-2019 Pandemic

**DOI:** 10.3389/fpsyg.2020.577886

**Published:** 2020-11-11

**Authors:** Leire Aperribai, Lorea Cortabarria, Triana Aguirre, Emilio Verche, África Borges

**Affiliations:** ^1^Department of Clinical and Health Psychology and Research Methodology, University of the Basque Country UPV/EHU, Donostia-San Sebastián, Spain; ^2^Department of Educational Sciences, University of the Basque Country UPV/EHU, Vitoria-Gasteiz, Spain; ^3^Department of Clinical Psychology, Psychobiology and Methodology, University of La Laguna ULL, San Cristóbal de La Laguna, Spain; ^4^Department of Psychology, School of Biomedical and Health Sciences, Universidad Europea de Madrid, Madrid, Spain

**Keywords:** COVID-19, mental health, physical activity, teacher, lockdown

## Abstract

The COVID-19 pandemic has led teachers to an unpredictable scenario where the lockdown situation has accelerated the shift from traditional to online educational methods, and relationships have been altered by the avoidance of direct contact with the others, with implications for their mental health. Physical activity seemed to be a factor that could prevent mental disorders such as anxiety or depression in this peculiar situation. Therefore, the aims of this study were to explore how teachers have been affected by the lockdown with respect to their mental health and their relationships in three main fields: work, family, and social relationships, and to know which is the role of physical activity in the mentioned variables. For that purpose, an online survey was designed to collect quantitative and qualitative data. Results showed that indoor physical activity acts as preventive in lockdown situations, whereas the level of activity does not affect mental health. Also, teachers have experienced higher levels of distress due to the workload generated during the lockdown. In conclusion, to prevent health problems among teachers in future similar situations, it would be important to facilitate the practice of physical activity at home. Furthermore, teacher training in blended or online educational methods would be crucial for their favorable work development.

## Introduction

The global expansion of the COVID-19 pandemic disease has carried out many consequences that may affect people's general health. On the one hand, the virus itself creates personal situations in which, in addition to the disease's symptoms, human emotions such as fear (Asmundson and Taylor, 2020), worry, panic, anxiety, or depression-related distress (Bao et al., 2020) can appear more commonly among people. Indeed, in recent studies about the psychological impact of this pandemic disease on the general population, an increase in depression and stress levels between the first days and the third week of the lockdown has been found (Ozamiz-Etxebarria et al., 2020; Rodríguez-Rey et al., 2020). Furthermore, anxiety has been related to impaired sleep in many studies (Rajkumar, 2020). On the other hand, social situations have changed due to the disease and the subsequent quarantine (Zhang et al., 2020), as well as due to attending to dependent or infected persons or those under other medical conditions at home or nearby. Also, it was due to the preventive measures applied by the government such as confinement or lockdown (Liu et al., 2020). In previous pandemics, individual differences seem to play an important role (Asmundson and Taylor, 2020). In any case, broader and more specific research of the impact on mental health is still needed (Mahase, 2020).

Moreover, people worldwide have found themselves coping with new professional scopes (Zhang et al., 2020). Some of them have completely stopped their work, and in brief, they will have to face their future with uncertainty; others have found their work hours increased and have managed risky situations (e.g., health and social workers, or product suppliers). This health crisis is also triggering an economic crisis at a global level and within a few weeks (UNESCO, 2020a).

Another factor influencing adults' personal, social, and professional fields is that related to the lockdown of children at home because, many times, parents have been involved in many roles and tasks at a time (Orte et al., 2020). Meanwhile, educational administrations have not stopped the scholar year, so that teachers have found themselves coping with online education at any level (Wang and Zhao, 2020) while attending to other personal issues. Furthermore, it should be mentioned that Spanish teachers' working conditions before this pandemic situation were already tight due to the teacher/student ratio from 25 to 36 per teacher (Education Youth Policy Analysis Unit in the Education Audiovisual Culture Executive Agency, 2020a) and the high amount of lessons (30–32 per week) they have to give (Education Youth Policy Analysis Unit in the Education Audiovisual Culture Executive Agency, 2020b). Also, all teachers should be prepared in all teaching roles for inclusive education and thus to work with all learners or students in individualized and close relationships, so that they must play a great role in a daily-based work and face-to-face with them. The work becomes even more difficult when this direct contact must be replaced by an online relationship, and many other factors should be considered. Teachers, in general, are not trained for e-learning programs and activities since this is not included in the curriculum of primary and secondary education (Education Youth Policy Analysis Unit in the Education Audiovisual Culture Executive Agency, 2020c). In addition, it should be emphasized that, in crisis situations, teachers may play an additional and crucial role. They can provide psychosocial support to learners. Firstly, teachers can create a safe and supportive interaction where students may express their emotions and experiences; secondly, they can include specific structured psychosocial activities in the teaching/learning process that can strongly help vulnerable students (Inter-Agency Standing Committee, 2007a). Therefore, teachers' workload can be considered quite high, and consequently, the teaching profession can be characterized by high levels of stress and physical complaints (Bogaert et al., 2014).

In the current situation, national governments all around the world are implementing new precautionary and responsive measures on a daily basis to contain the spread of the COVID-19 pandemic and to address this crisis that they have established a lockdown situation, social distancing advice, and educational measures such as temporary educational institutions' closures [European Agency for Special Needs and Inclusive Education, (n.d.)]. These global school closures are impacting over 60% of the world's student population, and in several countries, the implemented localized closures could impact millions of additional learners (UNESCO, 2020b). Moreover, school closures bring to people of many communities high social and economic costs, impacting mainly the most vulnerable and marginalized children and their families and exacerbating the already existing disparities not only within the education system but also in other aspects of their lives. Teachers also experience an important impact. Firstly, their students are concerned because of the interrupted learning and other collateral effects (disadvantages, lack of opportunities, poor nutrition, social isolation, or lack of care), and this makes even more difficult the teaching–learning process, mainly when parents are not prepared for distance and home schooling or they are not available to attend to their children. Secondly, teachers experience confusion and stress because they are often unsure of their obligations and how to maintain connections with students to support learning. Transitions to distance learning platforms tend to be messy and frustrating, even in the best circumstances. In many contexts, school closures lead to furloughs or separations for teachers. Thirdly, moving learning from classrooms to homes at scale and in a hurry presents enormous challenges, both human and technical (i.e., creating, maintaining, and improving distance learning, or measuring and validating learning) (UNESC, 2020c). In sum, from 1 day to the next, teachers have found themselves creating and managing virtual classrooms, communicating with their students and their parents over social media platforms, and learning by doing as they provide distance education to over 1.5 billion students affected by school closures all over the world due to the COVID-19 pandemic (UNESCO, 2020d). Despite governments' efforts to provide training and resources to support teachers in adapting to this new learning environment, turning from face-to-face to virtual classroom in such a short time has been a challenge as only a few teachers have strong digital and ICT skills. Therefore, in such unprecedented and uncertain times, it is normal for teachers to experience higher levels of stress and anxiety. Teachers need, indeed, socio-emotional support to face the extra pressure being put on them to deliver learning in a time of crisis (UNESCO, 2020d). Moreover, providing support for teachers' own psychosocial well-being is an essential component of supporting students (Inter-Agency Standing Committee, 2007b).

Nevertheless, the great changes in students', teachers', and parents' lives around the world caused by COVID-19 have brought to society an opportunity to test its capacity to adapt to sudden stressful situations in which people have been involved in new personal, social, educational, and professional environments and tasks. This health crisis will likely have long-term effects on education, so that it could become an opportunity to rethink the curriculum, teaching–learning assessment processes, and the development of students' competencies while strengthening their learning skills and sustaining their motivation. Moreover, the after-crisis period must be already previewed for the curriculum and learning continuity to be preserved (Daniel, 2020; UNESCO, 2020e).

This health and, consequently, economic crises caused by a pandemic that is reaching almost all countries in the world within a few weeks are unprecedented in the recent past. But lessons might be drawn from previous epidemics and economic crises (UNESCO, 2020a). It can be concluded from previous experiences that physical activity and exercise could help to mitigate the effects caused by the current pandemic on the mental and physical health of citizens worldwide. Being physically active should be highly recommended (Amatriain-Fernández et al., 2020) considering that physical activity could help in preventing psychological or mood disorders (Kwan et al., 2012) and improving the quality of life by decreasing the negative psychosocial effects of lockdown due to the COVID-19 pandemic (Slimani et al., 2020). In the same way, the role of physical activity in general health and well-being of teachers during lockdown should be important also, as it has been found that those teachers performing more exercise during leisure time, or in a more autonomous way, may prevent easier physical and mental health problems (Bogaert et al., 2014). In order to lead toward an after-crisis scenario and to prevent negative effects in future possible crises, it is worthy to know how these factors act in this lockdown situation. Therefore, this study aims to explore how teachers have been affected by the lockdown with respect to their mental health and their relationships in three main fields, such as work, family, and social relationships. Another objective followed by the study is to know which is the role of physical activity in the mentioned variables.

## Materials and Methods

### Method and Design

A mixed methods design, known as the third paradigm (Johnson and Onwuegbuzie, 2004; Denscombe, 2008), has been used. It is characterized for including in the same research both quantitative and qualitative methods, specifying in the design the weight and the sequence of each part and explaining how both approaches are linked (Creswell and Plano Clark, 2011). The applied design, the so-called concurrent triangulation, gives the same weight to qualitative and quantitative data (Smith et al., 2016).

### Participants

The sample of this research was composed of 345 teachers with a mean age of 44.62 years (*SD* = 9.53; 264 women; 80 men; 1 preferred not to say) currently teaching in Spain in primary and secondary education (see Table 1). Most of the teachers were working in public schools (n = 258), while 52 were in private schools and 35 in state-funded private schools.

**Table 1:** Participants' frequencies considering the educational levels in which they teach.

**Educational Level**	**Frequency**	**%**
Primary education (6–12 years old)	71	20.58
Secondary education (12–16 years old)	77	22.32
Postcompulsory secondary education (16–18 years old)	17	4.93
Others (i.e., languages, sports, arts)	53	15.36
More than one level	127	36.81

### Instruments

Data were collected using a questionnaire that included information about sociodemographic variables, teaching working conditions, and outdoor and indoor physical activities by using specific questions that were analyzed as quantitative variables. The Spanish version of the GHQ-12 (Sánchez-López and Dresch, 2008) was applied to measure mental health with the permission of the authors. This one-dimensional 4-point (0–3) Likert scale is composed of 12 items measuring aspects related to social dysfunction, anxiety, and depression. The questionnaire has acceptable psychometric properties, being its internal consistency acceptable (α = 0.76). Its external validity has been assessed by correlating with the ISRA's anxiety questionnaire, being the correlation with the whole scale medium (*r* = 0.57) and the correlation with the ISRA's factors high: Factor I *r* = 0.82; Factor II *r* = 0.70; and Factor III *r* = 0.75 (Sánchez-López and Dresch, 2008). Finally, open questions were applied to collect qualitative data about working conditions, family, and relationships.

### Procedure

Once the University of La Laguna's Ethics Committee's (CEIBA) approval [CEIBA2020-0401] and the permission of the authors of the Spanish version of GHQ-12 were obtained, a Google Form questionnaire with the mentioned sociodemographic, work, and physical activities' variables, the GHQ-12 scale, and the open questions (in this order) was created and sent to the participants. These were recruited by following a non-probabilistic snowball sampling procedure. For that purpose, social network was used, and corporate emails were sent in the first 3 weeks of the lockdown to Spanish teachers so that data were collected between the last week of April and the first week of May in 2020, during the lockdown and the 0 Phase when people had strong restrictions for outdoor activities and after 6 weeks that online education was established at all educational levels. All participants provided the informed consent to participate in the study. Therefore, the study fulfills the Declaration of Helsinki and the Organic Law 3/2018, of the 5th December, about Personal Data Protection and digital rights' warranty.

### Data Analyses

Firstly, descriptive statistics were performed. Secondly, Cronbach's alpha was estimated to analyze the reliability index of the GHQ-12 scale. Thirdly, to analyze the effect of physical activity with *t*-test, the participants were divided into two groups, differentiating between those who performed less and more physical activity. The first group was composed of those who practice little physical activity, that is, 3 h maximum per week; the second group was composed of those who practice more than 4 h of physical activity per week. Quantitative analyses were carried out by using the JASP software (JASP Team, 2020). Fourthly, quantitative information was triangulated to explore which were the best predictors of mental health by analyzing the contribution that the variables physical activity hours (outdoor and indoor) and teaching performance hours, as well as the number of students, have into the teachers' mental health with a Bayesian regression model. Fifthly, the perception that participants of both groups had about the changes that the lockdown situation had brought to the family, work, and social relationships was studied. Qualitative data obtained from the open questions were analyzed with the ALCESTE software (Lexical Analysis of Co-occurrences in Simple Text Statements; Reinert, 2001). This software uses statistical procedures to extract essential information from a text by receiving essential information, quantifying its strongest lexical structures, and grouping the co-occurrence, this last being the association by proximity of various words (nouns, adjectives, or verbs) using the chi-square statistic, with the aim of differentiating the most significant lexical words. Those words showing chi-squares higher than 3.841 were retained, following Camargo and Bousfield (2009) criterion. The analyzed unit is the elementary context unit (ECU), which corresponds to the idea of a sentence or a set of between 8 and 20 words (De Alba, 2004). One of the advantages of this approach is that it avoids the subjectivity involved in the construction of categories by the researcher, since the computer program establishes the connections using statistical procedures (Bauer, 2003).

## Results

### Descriptive Results of Teaching and Physical Activities and Reliability of the GHQ-12 Scale

On the one hand, participants admitted that they spent an average mean of 38.34 h (*SD* = 19.28 h) per week doing teaching activities. Regarding online activities, 64.34% (*n* = 182) manifested to have previously none or a little training in virtual teaching, and 56.81% (*n* = 196) participants claimed to do quite or much training on how to teach online during the lockdown.

On the other hand, in relation to physical exercise, 80% of the participants (*n* = 276) stated that they did physical exercise at home (*M*_Hours/week_ = 4.12; *SD* = 4.063), and 57.39% (*n* = 198) admitted that they went for a walk (*M*_Hours/week_ = 1.69; *SD* = 2.381).

Considering the teachers' general health, the average mean of the GHQ-12 total scores was 22.05 (*SD* = 5.26) (see descriptive statistics in Table 2). Moreover, the GHQ-12 scale showed an acceptable reliability index (Cronbach's α = 0.77). Finally, when comparing the GHQ-12 total scores between teachers doing high physical activity (*n* = 141; *M* = 21.596; *SD* = 5.426) and low activity (*n* = 204; *M* = 22.368; *SD* = 5.128), statistically significant differences were not found (*t*(343) = 1.34; *p* = 0.1809). The effect size was small (Cohen's *d* = 0.1468).

**Table 2:** Descriptive statistics of the GHQ-12 scale.

	**GHQTotal**	**GHQ1**	**GHQ2**	**GHQ3**	**GHQ4**	**GHQ5**	**GHQ6**	**GHQ7**	**GHQ8**	**GHQ9**	**GHQ10**	**GHQ11**	**GHQ12**
Mean	22.05	1.96	2.08	1.66	1.74	2.25	1.88	2.02	1.87	1.91	1.71	1.39	1.58
Std. Deviation	5.26	0.62	0.89	0.87	0.75	0.93	0.82	0.83	0.67	0.90	0.82	0.93	0.89

### Predictors of Mental Health: Bayesian Linear Regression

A Bayesian linear regression was carried out considering as predictors of the GHQ-12 score the amount of hours of physical activity performed at home, the hours dedicated to teaching performance, the number of students, and the number of hours spent walking away from home. An uninformed uniform prior [P(M)] of 0.063 was set for each possible model. The results suggest that the best regression model is the one including the time of physical activity at home and the hours dedicated to teaching work (BF10 = 11.07) compared to the null model. The regression coefficient for hours of physical activity at home is b_1_ = −0.096 and for teaching work b_2_ = 0.039. The constant of the model is b_0_ = 22.052. For instance, a teacher that is doing 5 h of physical activity a week and working for 39 h a week will have a mental health score of 21.9929 (see the equation below) measured with the GHQ-12.

$$\begin{array}{r} y=22.052+\left(-0.096\times \left[5-4.118\right]\right)\\ +\left(0.039\times \left[39-38.345\right]\right)=21.9929 \end{array}$$

### Perception of the Changes Experienced Due to the Lockdown Situation

The responses given about the changes found in the lockdown situation have been analyzed through the ALCESTE program in three areas: work, family, and social relationships. Considering that we have not found statistically significant differences in mental health between teachers with high and low levels of physical activity, and aiming to know what teachers from different levels say about the before mentioned areas, answers to three open questions have been analyzed on the basis of the high and low levels of physical activity, as presented below.

#### Question 1: Changes Observed by Teachers Regarding Their Professional Performance (“What Changes Do you Observe Regarding Your Professional Performance? How Do you Feel About It?”)

**Figure 1:**
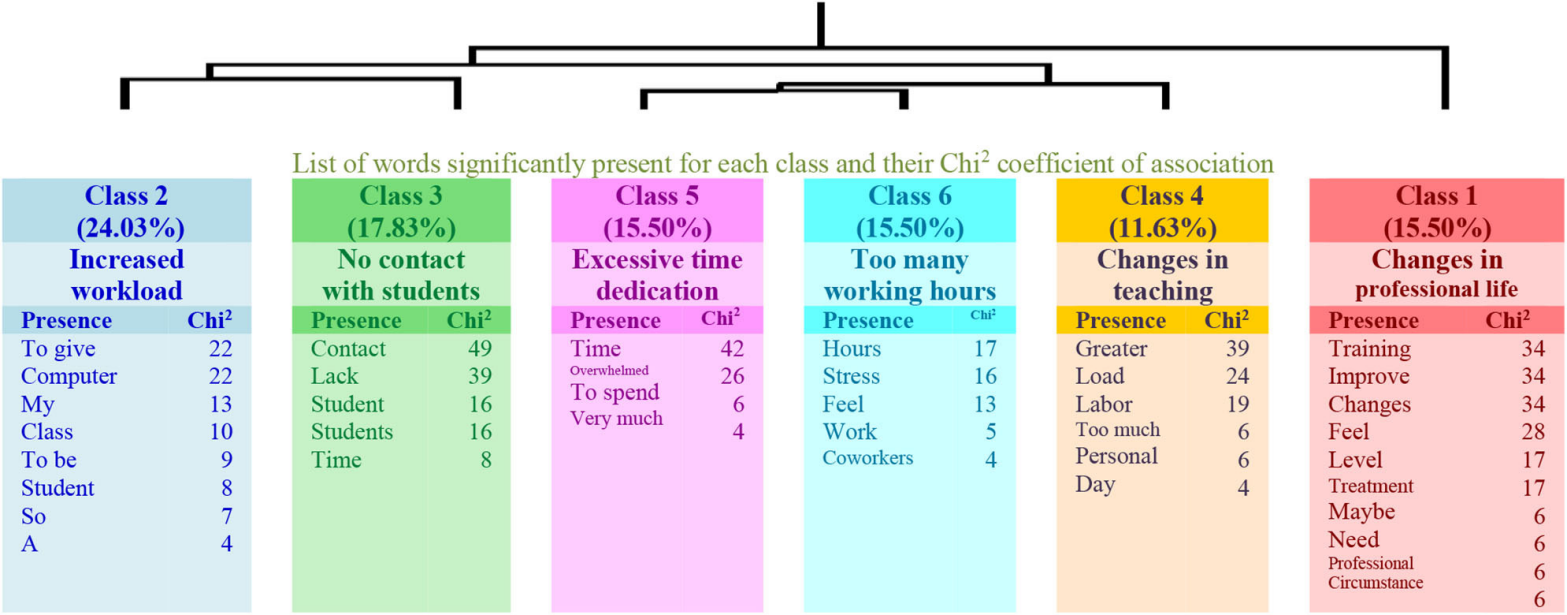
Dendrogram for Question 1: “What changes do you observe regarding your professional performance? How do you feel about it?” (Low physical activity).

On the one hand, in the analysis made for the low physical activity group, six factors or classes explaining 59% of the textual units were obtained (see the dendrogram in Figure 1). The first class (Changes in professional life) shows a more general content and connects with other two classes, 2 (Increased workload) and 3 (No contact with students), that are related to the way of teaching and to the contact with the students. The link between classes 2 and 3 connects with class 4 (Changes in teaching) and the link between classes 5 (Excessive Time Dedication) and 6 (Too many working hours). These classes have in common the shared complaint of working in excess and dedicating more hours due to the changes in teaching strategies.

**Figure 2:**
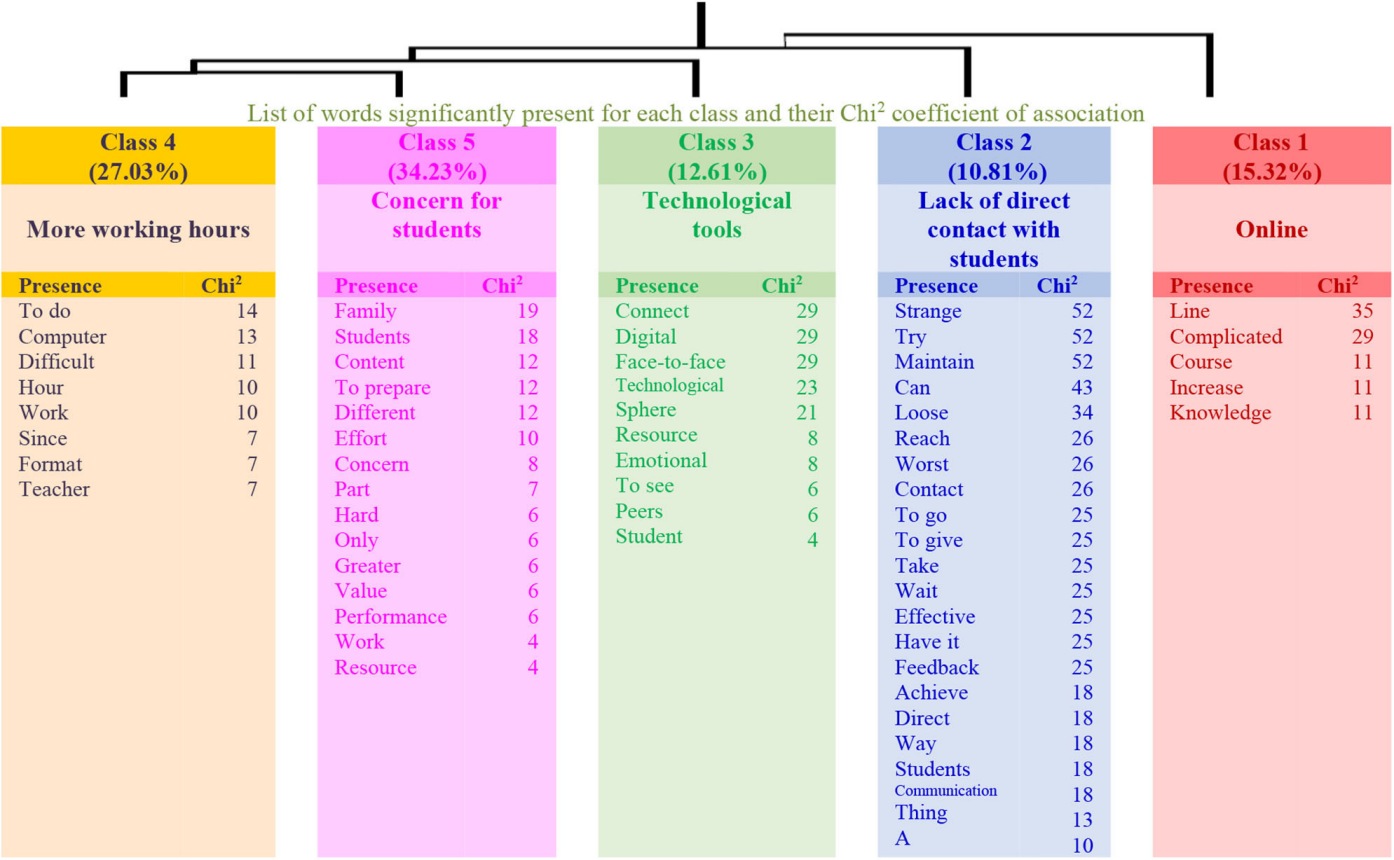
Dendrogram for Question 1: “What changes do you observe regarding your professional performance? How do you feel about it?” (High physical activity).

On the other hand, in the analysis made with the answers given by the teachers with higher levels of physical activity to the same question, a different structure emerges, this time composed of five factors or classes that explain 54% of the textual units but that are also organized in a more hierarchical structure. In this respect, the first class (Online) connects with class 2 (Lack of direct contact with students). The second connects with class 3 (Technological tools), and the third connects with the link of classes 4 (More working hours) and 5 (Concern for students). Therefore, teachers who have higher levels of physical activity express opinions about the consequences of online teaching during the lockdown, but these are weakly linked (see the dendrogram in Figure 2).

Table 3 presents the analyses carried out in the two groups, specifying the name of each class, the number of the elementary context units (ECUs) and their explained percentage, as well as the more representative word. The three examples with the highest χ^2^ are also shown for each class.

**Table 3:** Information of Question 1: “What changes do you observe regarding your professional performance? How do you feel about it?”

**Class**	**χ^**2**^**		**ECU**	**%**	**Word**
**Low physical activity group**					
**1**		*Changes in professional life*	20	15.50	Train *[Forma]*
Sentences	36	Obviously, the treatment with coworkers and students is different, I feel that I need them to improve as a professional, I also see this circumstance as a challenge in order to improve at a professional level *[evidentemente, eltratocon los compañeros y el alumnado es diferente, me siento quenecesitode ellos paramejorarcomo profesional, además veo estacircunstanciacomo un reto anivel profesionalparapodermejorar]*			
	30	Need for online training. Maybe we are closer *[necesidaddeformaciónonline.estamosmás unidos igual]*			
	28	All changes unleashed at a professional level are negative, because I feel that I have lost what gives meaning to my work: dealing with students *[loscambiosque ha desencadenado anivel profesionalson todos negativos, pues siento que he perdido lo-que dasentidoa mi trabajo: eltratocon el alumnado]*			
**2**		*Increased workload*	31	24.03	To give *[Dar]*
Sentences	11	The workload has increased greatly. In some groups the way of teaching is quite ineffective, so that they require a live and in-person explanation *[la carga de trabajo sehaintensificado muchísimo. lamaneradedar clasees bastante inefectiva en algunos cursos,yaquerequierende una explicación en directo y en persona]*			
	11	Care for my children, give more, prepare them more *[la atencióna misniños,darmás, prepararlos más]*			
	10	Full-time online teaching is an activity that requires being in front of the computer for 10 h a day. It is horrible *[la docenciaonline ajornada completa es unaactividadquerequiere estardelante delordenador10 horas diarias. es horroroso]*			
**3**		*No contact with students*	23	17.83	Contact *[Contacto]*
Sentences	15	Lack of contact with students *[falta de contacto conlos alumnos]*			
	14	Overflowed with so much work. There is no time limit. Given the lack of information, with uncertainty about how I will address the end of the course as a counselor *[desbordadacontanto trabajo. no hay límite horario.con incertidumbrerespectodecómo voy a abordar el finalde cursocomo orientadora dada lafalta deinformación]*			
	11	Isolation, less teamwork, lack of student contact. Sometimes frustrated *[aislamiento, menos trabajoenequipo,falta de contactoalumnado.frustradaa veces]*			
**4**		*Changes in teaching*	15	11.63	Greater *[Mayor]*
Sentences	17	Extension of the working day. Cancellation of days off, holidays. Exploited *[extensión de la jornada laboral. anulación dedíaslibres, vacaciones. explotado]*			
	17	More teamwork, generates a feeling of greater personal worth *[más trabajo en equipo, generandosensacióndemayorvalía personal]*			
	13	Greater labor disorganization and greater tension due to both legislative and lockdown time uncertainty *[mayordesorganizaciónlaboralymayor tensióndebido a la incertidumbre tanto legislativacomode tiempo de confinamiento]*			
**5**		*Excessive time dedication*	20	15.50	Time *[Tiempo]*
Sentences	23	I feel that I have to spend much more time. Overwhelmed *[siento que tengo-quededicarmucho más tiempo. agobiada]*			
	21	I keep spending a lot of time *[le sigodedicandomucho tiempo]*			
	13	I spend much more time. Overwhelmed by not having that time for personal issues *[empleo mucho más tiempo.agobiadapor no contar con esetiempo paratemas personales]*			
**6**		*Too many working hours*	20	15.50	Hour *[Hora]*
Sentences	7	More working hours *[máshorasde trabajo]*			
	7	I work 24 h. I started to dose them *[trabajolas 24 horas.estoyempezando a dosificarlo]*			
	7	I work many more hours and I feel that I cannot disconnect my work sphere from the family *[trabajo muchas más horas y siento que no puedo desconectar mi ambito laboral del familiar]*			
**High physical activity group**					
**1**		*Online*	17	15.32	Online
Sentences	107	I have been able to increase my knowledge about online courses. I feel good *[hepodidoaumentarmiconocimientoconcursos enlínea. me siento bien]*			
	67	Online education is complicated. Quiet *[locomplicadode la educación online. Tranquila]*			
	16	Online life, always at computer *[vida online, siempre ordenador]*			
**2**		*Lack of direct contact with students*	12	10.81	Strange [extraño]
Sentences	31	Not maintaining direct contact makes you see things differently, and you try to manage everything differently. It is a bit strange to teach children without being them in front of you *[no mantener contacto directohaceverlascosasde otra manera, y seintentagestionar todo demaneradistinta.resulta unpocoextraño dar clases a niños sin poder tenerloen frente]*			
	26	Not going to school is strange. Sometimes I feel like losing time, even when I try to maintain communication with the students, it is not even possible to reach 50(%) *[no ir ala escuela es extraño.A veceslo sientocomo tiempo perdido aun cuandoseintenta mantener comunicacióncon losestudiantes noselogra llegarni-siquieraa un50]*			
	18	Not being able to have contact with the students is what I take the worst. Feedback is not immediate, so you have to wait them to read and write, it is less effective for them *[elno poder tener contactocon los alumnos eslo-que peorllevo. elfeedback noes al momento, sino que hay queesperar aque lo lean y escriban, es menosefectivopara ellos]*			
**3**		*Technological tools*	14	12.61	Connect [Conecta]
Sentences	46	Obstacles for connecting emotionally with adolescent students and competitiveness among peers to see who is the most technological, intoxication due to digital resources *[obstáculos a la hora deconectaren elplano emocional conlosalumnosadolescentes y competitividadentre compañeros porver quién es el más tecnológico/ a, intoxicación derecursosdigitales]*			
	11	The development of the non-face-to-face session and trying to cover as much as possible with technological and strategic tools that allow me to achieve compliance effectively *[el desarrollo de la sesión nopresencialy tratando de abarcar lo más posibleconherramientastecnológicasyestratégicasque me permitan lograr cumplir de manera eficaz]*			
	9	Material resources and the face-to-face part *[recursosmateriales y la parte presencial]*			
**4**		*More working hours*	30	27.03	To do [Hacer]
Sentences	17	More hours at the computer, more working hours *[máshorasal ordenador, máshoras detrabajo]*			
	11	Complicated to correct activities since I am a plastic art teacher and the technical drawing exercises take me hours to correct when they could be corrected in much less time in print format *[me es muy complejo corregir las actividadesyaque soydeplástica y el dibujo técnico me llevahorascorregir ejercicios que se podrían corregir en mucho menos tiempode formaimpresa]*			
	11	More eyestrain since hours in front of the computer have increased *[más cansancio visualyaquehanaumentado lashorasdelante del ordenador]*			
**5**		*Concern for students*	38	34.23	Family [Familia]
Sentences	14	You work for more hours and sometimes you feel overwhelmed not only for preparing the classes, but for finding different resources to support a better understanding of the content and for helping families emotionally. Sometimes it is hard *[se trabaja más horasya veces tesientes agobiadapues nosolo prepararlas clases, sinobuscar distintosrecursosparaapoyar un mejor entendimiento de loscontenidos y ayudara lasfamiliasemocionalmente a veces es duro]*			
	14	My main concern is the assessment of students *[Mimayor preocupaciónes la evaluación de alumnado]*			
	8	The effort is not valued and it is better valued to be more a bum who sends and corrects a task and does not worry about students learning or doing well *[que no sevaloratodo elesfuerzo yque sevalora sermás un vago que manda una tareaysu correcciónyno sepreocupa porquesualumnadoaprendaoesté bien]*			

#### Question 2: Changes Observed by Teachers in Their Family Lives Due to the Lockdown (“What Changes do you See in Your Family Life? How do you Feel About it?”)

**Figure 3:**
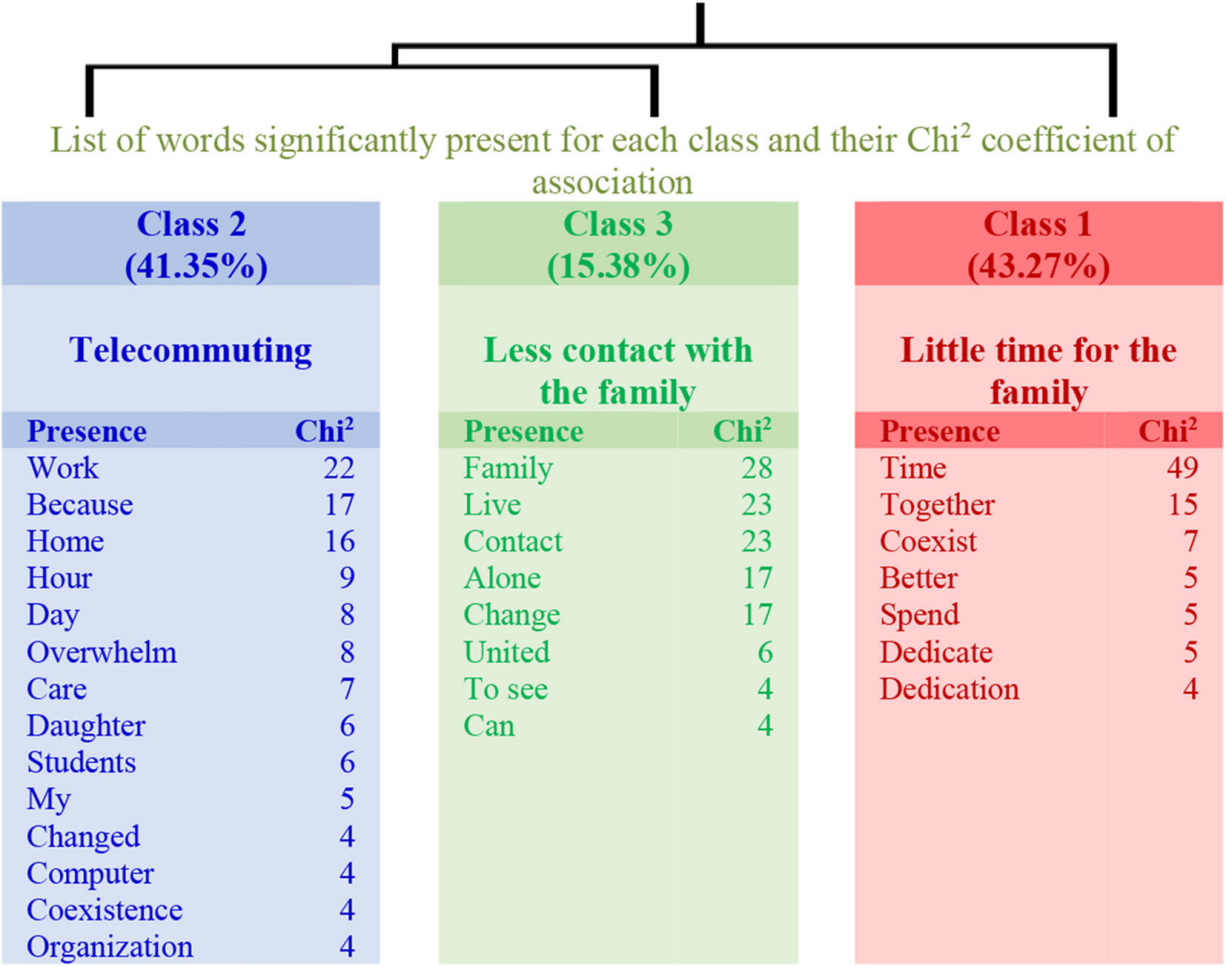
Dendrogram for Question 2: “What changes do you see in your family life? How do you feel about it?” (Low physical activity).

Teachers were asked about the changes they observed in family life as a consequence of the newly applied online teaching methods due to the pandemic. In the group with a low physical activity, a structure of three classes explaining 48% of the textual units emerges (see the dendrogram in Figure 3). The first class (Little time for the family) is connected to the link between the classes 2 (Telecommuting) and 3 (Less contact with the family). Therefore, teachers perceive a decrease in the contact with their family member that could be related to telecommuting and the consequent increase in the workload.

**Figure 4:**
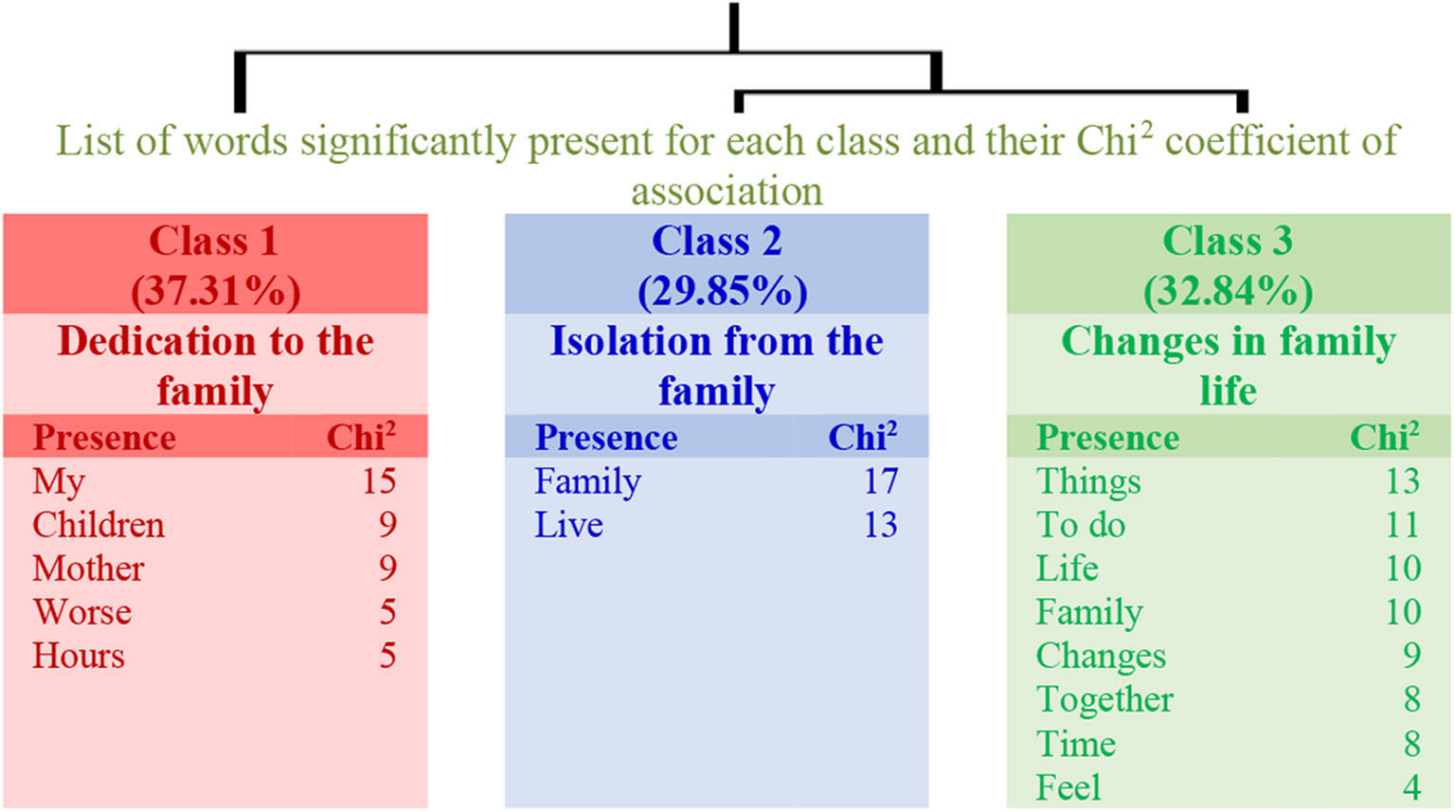
Dendrogram for Question 2: “What changes do you see in your family life? How do you feel about it?” (High physical activity).

Regarding those teachers with higher levels of physical activity, the changes observed in family life were explained in three classes with 45% of the textual units (see the dendrogram in Figure 4). Class 1 (Dedication to the family) connects with the link established by classes 2 (Isolation from the family) and 3 (Changes in family life). Thus, teachers with higher levels of physical activity manifest a decrease in the contact with family members but do not relate to the workload.

The results related to the analyses carried out regarding the changes observed in family life are shown in Table 4. The table specifies the name of each class, the number of the elementary context units (ECUs) and their explained percentage, as well as the more representative word. The three examples with the highest χ^2^ are also shown for each class.

**Table 4:** Information of Question 2: “What changes do you see in your family life? How do you feel about it?”

**Class**	**χ^**2**^**		**ECU**	**%**	**Word**
**Low physical activity group**					
**1**		*Little time for the family*	45	43.27	Time *[Tiempo]*
Sentences	5	We are more irascible and I have little time to dedicate to them. Thank goodness I do not have small children *[Estamos más irascibles y tengo pocotiempo paradedicarles. menos mal que no tengo hijos pequeños]*			
	5	Less time, we spend more time glued to the screen, I feel sad *[menos tiempos,pasamosmás pegados a la pantalla, mesientotriste]*			
	5	Much more busy and barely able to dedicate to them as much time as I would like *[mucho más atareado y sin poderles apenasdedicarlestodo eltiempoque me gustaría]*			
**2**		*Telecommuting*	43	41.35	Work *[Trabajo]*
Sentences	10	I have the impression that I do not take care of my daughter well because I am all day in front of the computer and I blame myself for it *[tengo la impresióndequenoatiendoamihija encondicionesporque estoytodo eldíadelante delordenador yme culpoporello]*			
	8	I'm almost all the day working, although we are all at home *[queestoycasi todo eldíatrabajando, aunque estemos todosencasa]*			
	8	Nothing has changed, only that we are working from home *[nadahacambiado, solo queestamos trabajando decasa]*			
**3**		*Less contact with the family*	16	15.38	Family *[Familia]*
Sentences	29	No change except not being allowed to see the whole family *[ningúncambio, excepto por nopoder vera toda la familia]*			
	29	I live alone. The only change is that I can not go on weekends to see my family *[vivosola. el únicocambioes q no puedo ir los fines de semana avera mi familia]*			
	11	Well, I live alone, thus it has little effect on my family unit *[bueno,vivo solay entonces afecta poco a mi unidad familiar]*			
**High physical activity group**					
**1**		*Dedication to the family*	25	37.31	My *[mis]*
Sentences	12	My children are always with me. Well *[mis hijosestán siempre conmigo. bien]*			
	11	I have more time to be with my children, but in worse conditions. it is very difficult to combine my work with my children *[tengo más tiempo paraestarconmishijos, pero en condiciones peores. hay muchadificultadpara compaginar mitrabajoconmishijos]*			
	9	Having a dependent mother, I lack hours during the day to combine professional and personal life *[al teneraunamadredependiente, mefaltan horasaldíapara compaginar vida profesionalypersonal]*			
**2**		*Isolation from the family*	20	29.85	Family *[Familia]*
Sentences	24	Need to see my family. I live on another island *[necesidad de ver a mi familia.vivo enotra isla]*			
	15	I can not see my family, I live alone and it is already beginning to weigh on me *[no puedo ver a mi familia,vivosola y ya está empezando a pesarenel ánimo]*			
	7	I live alone. I keep on touch with my family more than usual *[vivosola. mantengo mayorcontactodelhabitual conmi familia]*			
**3**		*Changes in family life*	22	32.84	Things *[Cosas]*
Sentences	10	Now we have time to do many things together. That feeling is fantastic *[ahora tenemostiempopara hacermuchascosasjuntos. esa sensación es fantástica]*			
	7	As for my family life I have had no changes *[en-cuanto-a mivida familiarnohetenido cambios]*			
	7	Many changes. I feel nostalgic of what we could do before lockdown *[muchos cambios.sientonostalgiadelo-que podiamoshacer antesdel confinamiento]*			

#### Question 3: Changes Observed by Teachers in Their Interpersonal Relationships (“What Changes do you See in Your Interpersonal Relationships? How do you Feel About it?”)

**Figure 5:**
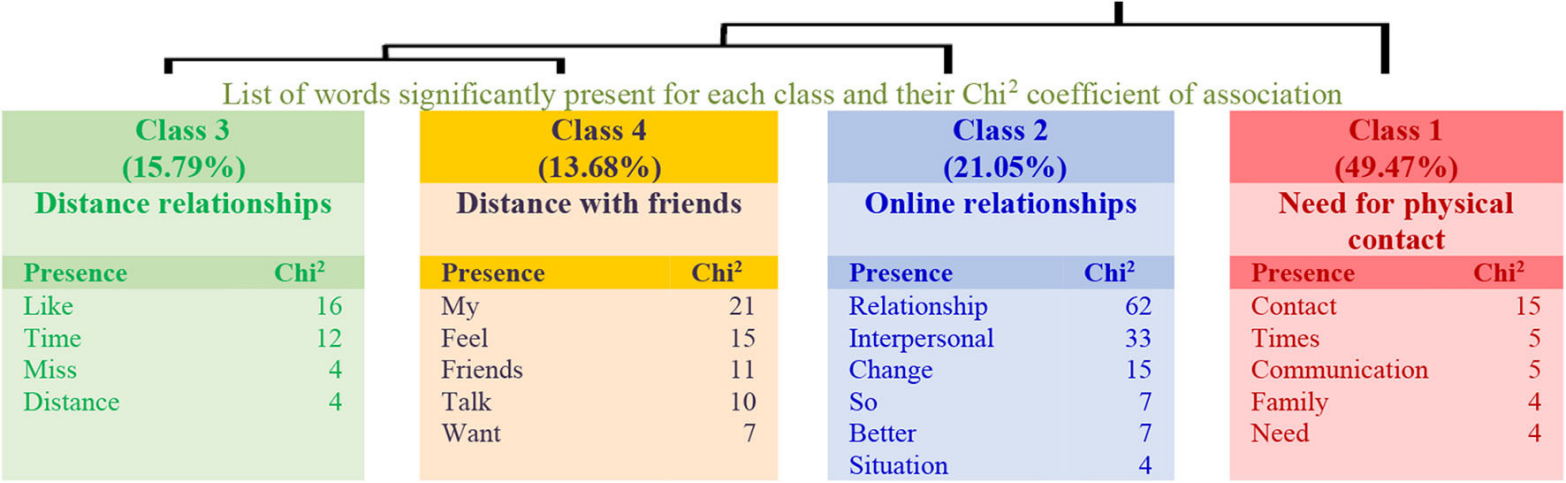
Dendrogram for Question 3: “What changes do you see in your interpersonal relationships? How do you feel about it?” (Low physical activity).

Regarding the answers to Question 3 in the group of lower levels of physical activity, four classes explaining 45% of the textual units have been obtained (see the dendrogram in Figure 5). In this structure, class 1 (Need for physical contact) connects with class 2 (Online relationships), and with the link between classes 3 (Distance relationships) and 4 (Distance with friends). Thus, teachers perceive distance despite the online relationship, and they miss physical contact in their relationships.

**Figure 6:**
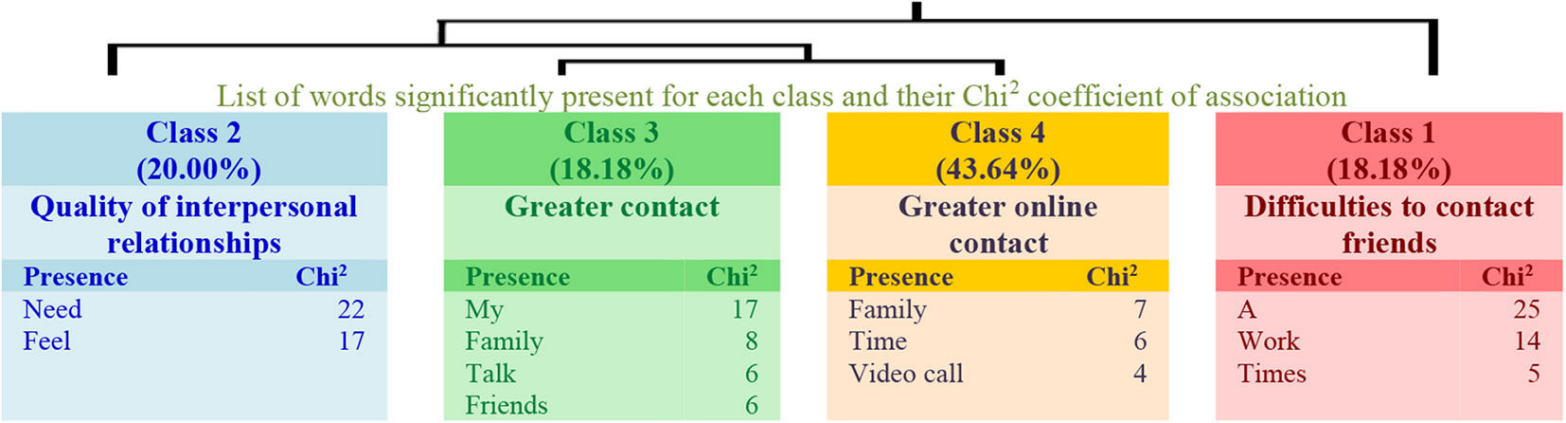
Dendrogram for Question 3: “What changes do you see in your interpersonal relationships? How do you feel about it?” (High physical activity).

As for the group with higher levels of physical activity, the answers are grouped into four classes explaining 38% of the textual units (see the dendrogram in Figure 6). In this structure, class 1 (Difficulties to contact friends) connects with class 2 (Quality of interpersonal relationships), and with the link between classes 3 (Greater contact) and 4 (Greater online contact). Therefore, teachers with higher levels of physical activity perceive greater contact but also difficulties and a loss of quality in their relationships.

Table 5 presents the detail of the analyses carried out in terms of the name of each class, the number of the ECUs and their explained percentage, and the more representative word, together with three examples of the highest values of χ^2^.

**Table 5:** Information of Question 3: “What changes do you see in your interpersonal relationships? How do you feel about it?”

**Class**	**χ^**2**^**		**ECU**	**%**	**Word**
**Low physical activity group**					
**1**		*Need for physical contact*	47	49.47	Contact *[Contacto]*
Sentences	14	I need physical contact *[necesito contactofísico]*			
	14	They are so virtual. I need physical contact *[son tan virtuales.necesitoelcontactofísico]*			
	14	It has been increased the contact via online and sometimes overwhelms me *[aumenta elcontactovía online y meagobiaa veces]*			
**2**		*Online relationships*	20	21.05	Relationship *[Relación]*
Sentences	15	What an interpersonal relationship. The only way we communicate is through diverse electronic media. whatsapp, mail, some facetime. I prefer the face to face relationship, this situation seems me very cold and unproductive *[querelacióninterpersonal. como único nos comunicamos espormedios electrónicos de diversa índole. whatsapp, mail, algún facetime. prefiero larelación cara acara, estasituaciónme parece muy fríaypoco productiva]*			
	7	I begin to consider the importance and need of some interpersonal relationships, to value some and lose interest in others *[empiezoaplantearme la importanciaynecesidad de algunasrelacionesinterpersonales,avalorar algunasydesinteresarmeporotras]*			
	7	Changes in relationships, they have become phone or videoconference relationships. Not totally satisfied, I prefer face-to-face relationships *[cambiosen las relaciones,hanpasadoaser telefónicaso porvideoconferencia. no totalmente satisfecha, prefiero presencial]*			
**3**		*Distance relationships*	15	15.79	To like *[Gusta]*
Sentences	21	I don't miss anyone. I like social distance. In fact, I don't care about social and nobody complains because it is normal *[no echode menos a nadie. megustaladistanciasocial. de hecho, paso de losocialy nadie se queja porque es lo normal]*			
	14	I would like to dedicate to them more time *[megustaríadedicarles más tiempo]*			
	10	Better. Because I have free time and I'm not overwhelmed by work and distance *[mejor. por-que tengotiempolibre ynoestoy agobiada por eltrabajoy la distancia]*			
**4**		*Distance with friends*	13	13.69	My [Mis]
Sentences	12	I feel further far away from my friends and colleagues. Lockdown is getting us away little by little *[mesientomás alejadade mis amigosy compañeros. el confinamiento nos está alejando poco-a-poco]*			
	12	Wanting to see my friends *[con ganas dever amisamigos]*			
	6	I feel good, I keep talking to both my friends and my girlfriend on a daily basis and I try to make video calls regularly *[mesientobien, sigohablandotantocon mis amigos como conmi novia a diario eintentohacer videollamadasconregularidad]*			
**High physical activity group**					
**1**		*Difficulties to contact friends*	10	18.18	A *[un]*
Sentences	18	Dependence on social networks. Not knowing for sure how to receive a written message in the working groups. Stressed by videoconferences *[la dependenciadelas redes sociales.nosaber con certezacomose recibeunmensaje escritoenlos gruposdetrabajo.estresada enlas videoconferencias]*			
	9	Communication is not easy. After working all day online, you don't feel like continuing to depend on an electronic device to talk to others *[noes fácil comunicarse. despuésde estartodo eldía trabajandoonlinenoapetece seguir dependiendode unaparato electrónico para hablar más]*			
	7	Sometimes a little far away from friends and parents because I don't visit them as much as I would like *[A veces unpoco alejadadeamigos y padres porquenolos visito tantocomoquisiera]*			
**2**		*Quality of interpersonal relationships*	11	20.00	Need *[Necesidad]*
Sentences	6	We value more those who have always been there and you need to see them. I feel nostalgic *[quevaloramosmás a quienes siempre han estado ahíytienes lanecesidadde verlos. me.sientonostálgica]*			
	6	Less and less contact, less joy and I feel sad *[cada vez menos contacto, menos alegríaymesientotriste]*			
	3	I feel good, because I am satisfied with my interpersonal relationships and the relationship remains the same, we remain the same *[mesientobien, porque estoy satisfecha con mis relaciones interpersonalesyla relación sigue siendo la misma, seguimos igual]*			
**3**		*Greater contact*	10	18.18	My *[Mis]*
Sentences	21	It is strange to talk to my neighbors or to someone apart from my partner and children. I miss my other family and friends *[se me hace extrañohablar con misvecinos oconalguien al margen de mi pareja e hijos. echo de menosami demásfamiliay amigos]*			
	15	I have more time to talk to my friends and I see more often my family, so this point has been positive *[tengo más tiempo parahablar con mis amigosy veo más a la familia, así-que este punto ha sido positivo]*			
	11	they have improved, I even talk to my parents more often than I ever did before *[hanmejorado, inclusohablo con mispadres más de lo que lo hacía antes]*			
**4**		*Greater online contact*	24	43.64	Family member *[Familiar]*
Sentences	10	More disagreements as there is much more contact and less time for outdoor activities *[más roce al haber mucho máscontactoy menostiempo paraactividades al aire libre]*			
	10	I make long calls with family and colleagues; these usually combine professional and personal aspects. We usually make a video call with friends, but personal direct contact is missed *[hago largas llamadas confamiliaresy con colegas; en estas se suelen compaginar aspectos profesionales y personales. con los amigos solemos hacer alguna videollamada, pero elcontactopersonal se echa de menos]*			
	6	I have more contact than before with some people thanks to the free time of both and the social networks *[hay personas con las que ahoracontactomás-que antes gracias altiempolibre de ambos y a las redes sociales]*			

## Discussion

The GHQ-12 scale measures state and not trait because items refer to how the participant perceives itself these days. In some studies, scores above 12 have been considered as indicating the existence of an emotional disorder (Ruiz et al., 2017). The average score of the sample (*M* =22.05; *SD* = 5.26) indicates symptoms of emotional problems. In case we were not experiencing the special situation of lockdown and the stress that this implies, a detailed clinical evaluation would be recommended.

These emotional problems are predicted negatively by the time devoted to physical activity weekly and positively with the number of hours working on teaching activity. Physical activity has been seemed to be a protector in developing emotional problems in this study and in previous (Kwan et al., 2012; Bogaert et al., 2014; Amatriain-Fernández et al., 2020), but in this study, the level of activity in general does not make the difference, while it seems that the type of activity, specifically indoor physical activity, explains part of the variance on mental health; thus, it should be enhanced in order to improve teachers' mental health. However, other possible stressors such as the number of students seem to have no relationship with mental health. Nonetheless, the predictor power of these variables is weak, so other variables should also be considered to be studied as predictors of mental health in teachers.

The results extracted from the qualitative responses show differences related to the observed changes in teachers' lives due to lockdown. Clear differences are observed in the discourse of the two groups of teachers. On the one hand, those who report having low physical activity point out changes in their professional lives and in their relationship with students and focus on showing their concern for the greater dedication and longer working time required by online teaching. On the other hand, teachers who have more time to develop physical activity show a greater dispersion in their responses. In this respect, two classes are related to their relationship with students, the other two have to do with changes or challenges in their teaching performance (the change to online teaching and the need to master technological strategies), and the last one is related to the expression of opinions regarding the increased workload. This aspect has already been included in the literature, since telecommuting is more demanding in hours, due to the fact that the environment does not change, along with having to put into play new skills that they were lacking on a regular basis (Santillán, 2020).

Despite the fact that work changes determinate modifications in family life, which are included in two classes in the low physical activity group and in three classes in the other group, in both groups, to spend more time with the family has been considered as a positive indicator. In other words, both groups regret the difficulty they have to meet with the family due to the lockdown.

Social relationships have also been affected by the lockdown. In this section, the opinions expressed by the group with greater physical activity are more optimistic than those manifested by the group with little physical activity. In the first group, the distance feeling is mentioned only in one class, while the other expressions indicate a positive attitude, considering the value of maintaining online relationships. Meanwhile, the group with lower levels of physical activity manifests complaints related to the lack of relationships in all classes. Further research should be addressed to know more about the type of physical activity or other variables that improve mental health.

The main limitation of this work is that it has not been possible to cover a more specific regional and a broader international perspective. Another limitation is that other variables related to working and personal conditions during the lockdown should be addressed to assess their impact on mental health, aspects that were not afforded due to length limitations of the study. The last limitation to be considered is that classes explained a medium percentage of the textual units. Thus, these results should be confirmed with further research.

In summary, this situation of lockdown has led to major problems in teachers' lives, as evidenced by the pressure that online educational methods have placed on them: many hours of work and difficulties due to the lack of physical contact or due to the obstacles created on combining personal life with family. Obviously, we will have to learn from this experience in several ways. On the one hand, it is essential to study which digital competences both teachers and students have, as well as parents, since in the vast majority of cases, they have had to act as a bridge to facilitate the teaching–learning process of their children (Cuetos et al., 2020). This is already invariable whether the health requirements force a new lockdown or not. On the other hand, the situation created by the COVID-19 pandemic disease has evidenced the advantages of online training and its drawbacks because it can help in expanding borders and bringing education to every home. But for this to become possible, many limitations, mostly technical (lack of computers, not enough for all family members, inadequate or non-existent internet connections), have to be overcome as teachers' concerns have manifested, which will have to be taken into account by universal digitization policies, by the rulers, and by public policies that prevent the digital gap. Other limitations could be those related to knowledge and skills, and teachers have mentioned that they might have to be trained in the didactic and instructional value that each resource and each strategy has, since there is not a direct translation from what is done in the classroom to what has to be done online. Considering this difficult situation, it is also necessary to design better-structured teacher training plans, which do not generate an excessive workload, as it has been reflected in the results of this research. Obviously, no one was prepared to make the leap from classroom to online teaching from 1 day to another.

Another point that should be considered is that in some countries, as in the case of Spain, the hardest moment of lockdown prevented from leaving home except for very justified reasons. Therefore, it is not surprising to find out low physical activity scores. Given the importance that physical activity has in mental health (Fuentes-Barria et al., 2018), it would be convenient to establish support programs to encourage physical activity for similar situations in the future, in the case that health requirements force citizens to return to lockdown.

In conclusion, the hard lesson that has involved alleviating the difficult situation of the pandemic disease leads to three action points: the establishment of measures to facilitate the online teaching resources; the design of teaching strategies that favor teaching–learning processes based on blended or online methods; and the development of support programs to foster physical activity among citizens.

## Data Availability Statement

The datasets presented in this article are not readily available because we didn't ask participants any permission and informed consent to share the data. Requests to access the datasets should be directed to taguirre@ull.edu.es.

## Ethics Statement

The studies involving human participants were reviewed and approved by Universidad de La Laguna ULL. The patients/participants provided their written informed consent to participate in this study.

## Author Contributions

Authors in this manuscript contributed as stated in this section. On the one hand, LA, LC, TA, and ÁB were involved in the conceptualization of the project and in the acquisition of the data. On the other hand, EV and ÁB were involved in the analysis and interpretation of the data. Finally, all authors were involved in the drafting and revision of the work for intellectual content, provided approval for submission of the contents for publication, and agreed to be accountable for the accuracy and integrity of the project.

## Conflict of Interest

The authors declare that the research was conducted in the absence of any commercial or financial relationships that could be construed as a potential conflict of interest. The reviewer NI declared a shared affiliation with several of the authors, LA, LC, to the handling editor at time of review.
